# Population genetic structure between Yap and Palau for the coral *Acropora hyacinthus*

**DOI:** 10.7717/peerj.2330

**Published:** 2016-08-18

**Authors:** Annick Cros, Robert J. Toonen, Sarah W. Davies, Stephen A. Karl

**Affiliations:** 1 Hawai‘i Institute of Marine Biology, University of Hawai‘i, Mānoa, Kāne‘ohe, HI, United States; 2 Department of Integrative Biology, University of Texas, Austin, TX, United States; 3 Department of Marine Science, University of North Carolina, Chapel Hill, NC, United States

**Keywords:** Genetic connectivity, Gene flow, Coral colonization, Reef conservation

## Abstract

Information on connectivity is becoming increasingly in demand as marine protected areas are being designed as an integral part of a network to protect marine resources at the ecosystem level. Larval dispersal and population structure, however, remain very difficult to assess. Here, we tested the predictions of a detailed oceanographic connectivity model of larval dispersal and coral recruitment within Palau and between Palau and Yap, which was developed to support the review of the existing network of marine protected areas in Palau. We used high throughput microsatellite genotyping of the coral *Acropora hyacinthus* to characterize population genetic structure. Pairwise *F*′_*ST*_ values between Palau and Yap (0.10), Palau and Ngulu (0.09) and Yap and Ngulu (0.09) were all significant and similar to pairwise *F*′_*ST*_ values of sites within Palau (0.02–0.12) and within Yap (0.02–0.09) highlighting structure at island scale and indicating that recruitment may be even more localized than previously anticipated. A bottleneck test did not reveal any signs of a founder effect between Yap and Palau. Overall, the data supports the idea that recovery of *A. hyacinthus* in Palau did not come exclusively from a single source but most likely came from a combination of areas, including sites within Palau. In light of these results there seems to be very little connectivity around the barrier reef and management recommendation would be to increase the number or the size of MPAs within Palau.

## Introduction

Over the past forty years, the United Nations Environment Programme (UNEP) Regional Seas Convention and Action Plans has been encouraging countries to work together to protect marine resources at a regional scale (Johnson et al., 2014). In the last decade, this approach targeting the protection of coral reefs has been adopted through initiatives such as the Micronesia Challenge, the Coral Triangle Initiative on Coral Reefs, Fisheries and Food Security, and the Caribbean Challenge Initiative. Island nations are joining forces to protect their near-shore resources, not only at a national scale but also by considering the integrity of their ecosystems at a regional scale. As a consequence, managers have had to scale up existing conservation strategies to adapt them to larger areas. One approach has been to develop Marine Protected Area (MPA) networks as a tool to address the conservation of coral reefs across borders. Designed to maintain connectivity at the scale of ecosystem processes, MPA networks build resilience by spreading risk in the case of localized disasters, climate change, failures in management or other hazards, and overall, help protect biodiversity and fisheries resources (IUCN, 2008; Walton et al., 2014). These networks also allow for building upon existing MPAs by maintaining structures that are already in place and enhancing resilience to these areas (McLeod et al., 2009).

As a member of the Micronesia Challenge, Palau is one of the nations to have adopted resilient MPA networks as part of their strategy to effectively conserve at least 30% of the near-shore marine resources across Micronesia. Palau is an archipelago that suffered heavy bleaching mortality during the 1998 El Niño bleaching event and in response to that mortality, established a network of marine protected areas to encourage reef recovery. The initial MPAs, however, were not designed with the specific purpose of maintaining connectivity between reefs and there has been a national effort to review the design of their MPA network to provide regional resilience to both local and global scale stressors (Golbuu et al., 2005).

In practice, implementing MPA networks that are interconnected and thus resilient faces the challenge of understanding connectivity of marine communities. Many marine organisms reproduce via minute pelagic larvae that are difficult to track and the barriers and drivers influencing their dispersal are not always obvious, making information on connectivity difficult to determine (reviewed in Levin, 2006; Cowen & Sponaugle, 2009). To address such issues, researchers have taken two main approaches: first, developing oceanographic models of the dispersal of particles forced with physical data (e.g., wind and tides) or second, indirect/direct tagging via molecular (i.e., DNA) or chemical markers (i.e., otolith/statolith chemistry) to study gene flow between populations (Hellberg, 2007; Cowen & Sponaugle, 2009).

In the most detailed oceanographic connectivity model for Palau to date, Golbuu et al. (2012) incorporated reef architecture at 500 m scales with over 30 years of oceanographic data to predict larval dispersal and coral recruitment both within Palau and between Palau and Yap, a neighboring Micronesian archipelago 452 km from Palau. They concluded that Palau recovered quickly after the mass 1998 bleaching mortality due to a pulse event of larval dispersal from Yap in 1999. The model also predicted considerable local retention at all sites in the Palau archipelago on a short time scale (<3 days), which changed at longer time scale (>3 days) with the northern lagoon showing the most flushing. The model also indicated a directional dispersal from densely populated areas (i.e., the southern lagoon) to less densely populated areas (i.e., the northern lagoon). Based on the results of their dispersal modeling, Golbuu et al. (2012) recommend building a network of MPAs at a regional and national scale that would link Yap and Palau, and Palau’s northern and southern reefs.

A common alternative to an oceanographic model to estimate rates, distances and patterns of dispersal is genetic analysis. Microsatellites have been the marker of choice, using fragment analysis to create genotypes based on length. This method, however, has several issues, including the problem of homoplasy, which can reduce allelic diversity in populations and inflate estimates of gene flow when mutation rate is high (Selkoe & Toonen, 2006). Sequencing microsatellites can resolve this issue by allowing scoring microsatellites according to sequences, yet very few studies have tried this approach. Here, we use high throughput microsatellite genotyping of the coral *Acropora hyacinthus* to characterize population genetic structure between Yap and Palau to test the predictions made by the dispersal modeling of Golbuu et al. (2012).

## Methods

### Study species

*Acropora hyacinthus* was an abundant tabular coral found on the reef slopes of Palau prior to the 1998 bleaching event. During their 2001 assessment, Bruno et al. (2001) estimated a near complete loss of this coral in the areas they sampled. By 2005, Golbuu et al. (2007) observed that the same species was dominant in the shallow reef slopes, raising the question of where the larvae originated from to allow for such a successful recovery and thereby making *A. hyacinthus* and ideal candidate to study coral connectivity.

**Figure 1:**
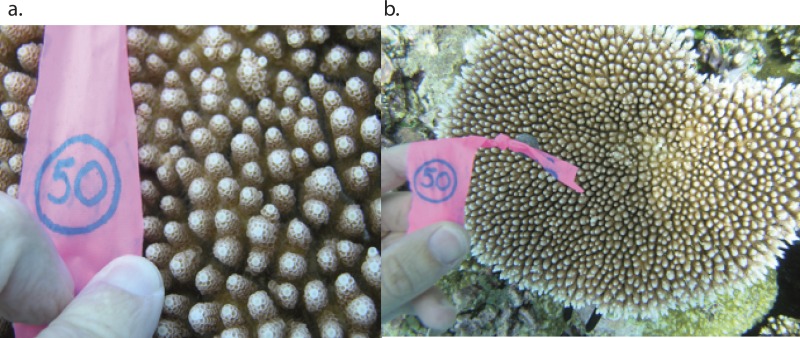
Example of a colony of *Acropora hyacinthus* collected in Palau.

*A. hyacinthus* is usually found between 3 and 10 m deep on barrier reefs and is readily identifiable by the rosette formation of its calices (Fig. 1). *A. hyacinthus* is a hermaphrodite broadcast spawning coral that produces feeding larvae (Toh, Peh & Chou, 2013) with a pelagic larval duration of approximately 90 days under laboratory conditions (Márquez et al., 2002). Little is known, however, about the pelagic duration or swimming behavior of larvae in the field, which makes realistic incorporation of biological parameters into oceanographic models difficult (Paris, Chérubin & Cowen, 2007; Woodson & McManus, 2007).

### Sampling locations and methodology

**Figure 2:**
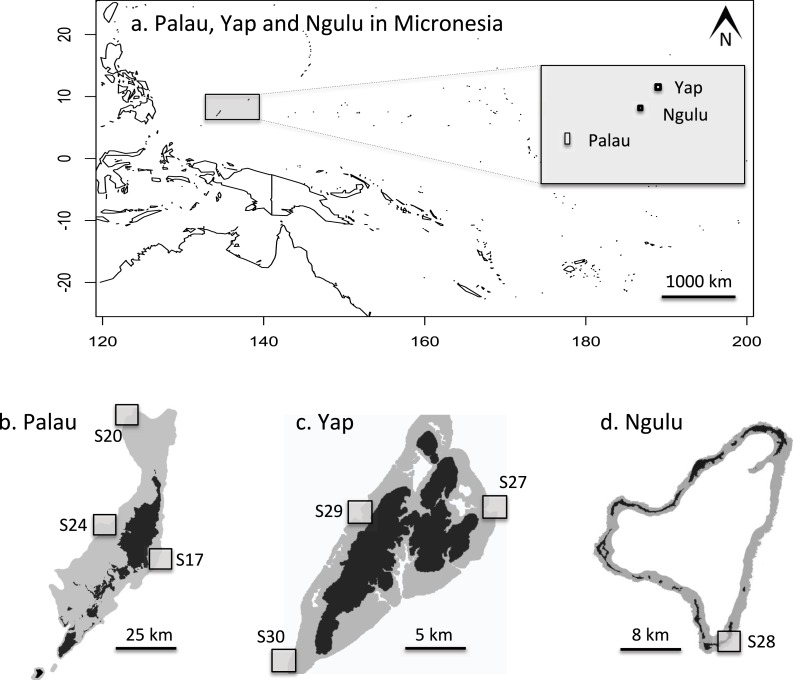
Maps of sampling locations: (A) overview of location of Yap, Ngulu and Palau in Micronesia; (B) sample sites in Palau; (C) sample sites in Yap; (D) sample sites in Ngulu.

In May 2012, three sites on the outer barrier reef of Palau were sampled at a shallow depth (<10 m) using SCUBA (Figs. 2A and 2B). Sites were selected to represent a range of habitats, exposures and management categories found on the barrier reef: S17 “West Palau” within a fully protected no-take area on the west side, S20 “North Palau” at the tip of the northern lagoon in a less strictly protected area and S24 “East Palau” on the east coast in a reef impacted by anthropogenic stressors (Golbuu et al., 2012). At each of these three sites, 48 colonies of *A. hyacinthus* were collected haphazardly in a 4 × 200 m belt transect, for a total of 144 colonies from Palau. One small branch tip (<1 cm) was cut and preserved in salt-saturated DMSO at room temperature (Gaither et al., 2010). In addition, a total of 132 samples were collected from three different sites around Yap, and another 46 colonies were sampled from a single site at Ngulu as described in Davies et al. (2015, Table 1, Figs. 2A, 2C and 2D). Importation was permitted by the Convention on International Trade in Endangered Species of Wild Fauna and Flora permit #FW 12-091.

**Table 1:** GPS coordinates, main island group and number of samples genotyped for each site.

Site	Island	GPS	Number
S17 East	Palau	7°025′46.92N, 134°038′31.272E	48
S20 North	Palau	8°000′05.04N, 134°032′09.960E	48
S24 West	Palau	7°031′50.52N, 134°024′03.960E	48
S27 Goofnuw Channel^a^	Yap	9°034′26.40N, 138°120′19.200E	37
S29 West Outer Reef^a^	Yap	9°033′47.30N, 138°050′71.500E	48
S30 South Tip Reef^a^	Yap	9°026′05.40N, 138°020′10.400E	48
S28 Ngulu^a^	Ngulu Atoll	8°180′12.00N, 137°290′18.700E	43

**Notes.**

a Samples previously collected by Davies et al. (2015).

### DNA extraction and sequencing

For colonies from Palau, Genomic DNA was isolated following the DNeasy 96 Blood & Tissue Kit (Qiagen, Valencia, CA, USA) protocol. Two sites with 48 colonies each were extracted on a 96 well plate. For each colony, a 2 mm^3^ piece of coral from the tips of one branch was ground and incubated overnight at 55 °C in 180 µl of Qiagen Lysis buffer and 20 µl of Qiagen Proteinase K (600 mAU/ml). DNA was eluted in 200 µl of PCR grade water, with 100 µl frozen until amplified and 100 µl saved for future use at −20 °C. A sample of 50 DNAs was quantified on a SpectraMax (Molecular Devices, Sunnyvale, CA, USA) absorbance microplate reader using Accuclear Ultra High Sensitivity quantitation Kit (Biotium, Hayward, CA, USA). Quantities of DNA were very similar for all colonies, ranging from 5 to 10 ng/µl. The DNA of colonies originating from Yap and Ngulu were isolated according to Davies et al. (2015).

Eighteen microsatellite loci developed by Wang, Zhang & Matz (2009) were selected for this study (Table S1). We designed short tags according to  Faircloth & Glenn (2012) to create 48 unique colony IDs on the forward primer for each microsatellite locus (Table S2). Polymerase chain reactions (PCRs) for each colony were performed in a 25 µl reaction volume containing 12.5 µl of MyTaq™ Red Mix (Bioline, Taunton, MA, USA), 1 µl of each forward and reverse primer at 5 µM, 1 µL of genomic DNA (5–10 ng/µl) and 9.5 µl of water. Thermal cycling followed a touch-down protocol with an initial denaturation at 95 °C for 3 min followed by 20 cycles of 95 °C for 15 s, 63–55 °C for 15 s (annealing temperature reduced 0.4 °C each cycle), 72 °C for 30 s followed by 20 cycles of 95 °C for 15 s, 55 °C for 15 s, 72 °C for 30 s with a final elongation at 72 °C for 3 min.

Two µl of each uniquely barcoded amplicon were pooled by site for subsequent library construction and sequencing. The pooled samples were concentrated by reducing the total volume using Amicon Ultra 0.5 mL centrifugal filters (EMD Millipore, Darmstadt, Germany) and cleaned using Agentcourt Ampure XP (Beckman Coulter Inc., Brea, CA, USA) to eliminate traces of dye and unincorporated dNTPs and primers. Genomic libraries were generated using the KAPA Hyper Prep kit (Kapa Biosystems, Wilmington, MA, USA). A unique Illumina adaptor (Illumina Inc., Hayward, CA, USA) was ligated to each pool of individually barcoded amplicon samples, creating a site-specific tag (ID) and generating the following unique structure: siteID-colonyID-FWDprimer-flankingregion-tandemrepeats-flankingregion-RVSprimer. Libraries were sequenced on an Illumina MiSeq at the Hawai‘i Institute of Marine Biology (HIMB) Evolutionary Genetic core facility.

### Bioinformatics (Table S3)

#### Low quality trimming

Illumina adaptors and barcodes were removed and sequences were demultiplexed by site through the Illumina MiSeq Reporter (MSR) software (Illumina, Inc). Forward and reverse sequences were merged using pear (Paired-End reAd mergeR) (Zhang et al., 2014). Sequences were then further demultiplexed according to primer and colony barcodes using integroomer (M Belcaid, 2015, unpublished data, http://courge.ics.hawaii.edu/inte/groomer/). Low quality sequences and reverse primers were trimmed using trimmomatic (Bolger, Lohse & Usadel, 2014). Cleaning resulted in sequences consisting of flanking region, tandem repeats and flanking region. Identical sequences were then collapsed into unique sequences and counted (=depth; the number of times a given sequence was repeated in the library).

#### Filtering

A set of filters developed in python (https://github.com/annickcros/Ahyacinthus-filters.git) was then applied to the sequences to eliminate PCR and sequencing artifacts. A length filter was applied to eliminate sequences that were less than 15 base pairs and sequences that were greater than 190 base pairs. This maximum length was determined by adding 4 additional repeats to the longest microsatellite sequence in the data set (Table S1). The file with the rejected sequences was checked by eye. None of the sequence rejected were true microsatellites. Two depth filters were applied to the sequences of the entire dataset, which were pooled by locus. The first eliminated any sequences with fewer than 10 reads the second eliminated any sequence that was present in less than two individual colonies regardless of sequencing depth (see below).

#### Scoring

To score microsatellite alleles, for each colony at each locus, the sequence with the highest depth was initially selected. Any sequence that had a depth greater than half of the depth of the most frequent sequence was also selected. This approach resulted in colonies with one (homozygote), two (heterozygote), or in some cases, more than two alleles per locus (see below).

#### More than 2 alleles

After filters and scoring, there were still a large number of colonies that had more than two apparent alleles among the sequence reads (i.e., multiple sequences were represented at least half as often as the most common allele in the amplicon library). These alleles typically varied either by a single nucleotide difference or by an indel in the repeat region, and could have derived from somatic mutations, individual chimerism, PCR or sequencing errors, or some combination of these factors.

For colonies in which more than two alleles passed the initial filters, we selected the two alleles used for analysis in two different ways. In both cases, we first selected the sequence with the highest depth as the first allele. The second allele was then selected either by: (1) the allele with the next highest sequencing depth (selection by depth), or (2) at random from all the available alternative choices (random selection). In a few cases of selection by depth, there were multiple alternative alleles that were equally frequent, therefore the second allele was selected at random from among the tied second-highest depth reads. Random selection with replacement of the second allele was repeated 10 times to generate 10 independent files.

### Splitting flanking region sequences and simple tandem repeats

We separated simple tandem repeats (STR) from their flanking regions using emboss: etandem (Rice, Longden & Bleasby, 2000) and created genotypes based on the STR length.

### Analysis

#### genodive files

Datasets were converted to genodive v. 2.0b27 (Meirmans, 2009) file format. Individual genotypes were created using two different methods. First, we used sequence length similar to peak calling in a microsatellite fragment analysis, such that all sequences of the same length, regardless of underlying sequence variation, would be scored as the same allele (sequence length). Second, we identified alleles by their sequence (ID) so that only two exactly identical alleles had the same ID, whereas alleles with the same length but differing in nucleotide composition would have different IDs (unique ID).

Only loci with less than 15% missing data and colonies with less than 35% missing data were used for the analysis. The final dataset analysis was carried out on 11 loci (Table S3). The final number of colonies for each locus for each site varies between 37 and 48. The number of allels and allelic richness is reported in Table 2.

**Table 2:** Alleles and allelic richness per locus and per site. Number of alleles (in white) per locus and per site. Allelic richness (in grey) based on 24 colonies per locus and per site.

	Site 17		Site 20		Site 24		Site 27		Site 28		Site 29		Site 30		Total	
loc1	2	2.00	2	2.00	2	2.00	2	2.00	2	2.00	2	1.99	2	2.00	2	2.00
loc3	5	3.94	5	4.74	6	5.50	5	4.99	6	5.52	6	5.49	6	5.46	7	5.27
loc4	15	12.75	18	15.49	13	11.51	13	12.51	16	13.71	13	10.97	16	13.99	21	14.88
loc5	8	6.77	6	5.46	6	4.53	4	3.88	7	5.38	6	4.50	4	4.00	10	5.09
loc6	2	2.00	3	2.97	4	3.98	4	3.98	4	3.04	3	2.56	4	3.49	4	3.46
loc8	10	8.37	11	9.55	13	12.14	9	8.51	13	11.22	11	10.30	13	11.36	15	10.99
loc11	7	6.02	5	4.96	7	6.48	7	6.55	5	4.78	6	5.91	7	6.72	8	6.65
loc12	13	10.27	17	13.83	12	10.94	13	11.77	12	10.93	11	9.35	14	11.51	20	11.69
loc13	5	4.25	5	4.75	6	5.36	7	6.59	4	3.52	5	4.75	6	5.79	7	5.41
loc14	8	6.25a	7	6.20	11	9.39	6	5.48	8	7.26	10	8.40	5	4.54	13	7.69
loc16	1	1.00	2	1.98	6	4.92	5	4.78	2	2.00	5	4.38	6	4.99	7	4.15

#### Comparison of datasets

To compare the different datasets created by alternate strategies of filtering and scoring, we looked for bias in population differentiation using AMOVA in genodive to calculate *F*_*ST*_, *F*′_*ST*_ and *F*_*IS*_ and corresponding significance values among and within populations. Indices of genetic diversity, including observed heterozygosity (*H*_*O*_), expected heterozygosity within populations (*H*_*E*_), corrected total heterozygosity (*H*′_*T*_) and Nei’s corrected fixation index *G*′_*ST*_, were used to quantify the population diversity (Table 2) and check for any potential bias between datasets created using different strategies.

#### Population differentiation

We carried out an AMOVA to calculate population differentiation by island groups. Using Genodive v. 2.0b27, we carried out two pairwise differentiation tests (20,000 permutations). The first was among islands. The second pairwise differentiation tests were carried out among all seven sites and exact tests of population differentiation (100,000 steps) were carried out using genepop v. 4.4.3 (Raymond & Rousset, 1995; Rousset, 2008) to report the *p* value of the pairwise *F*′_*ST*_ (Table 3).

**Table 3:** *F*′_*ST*_ (above the diagonal) and significance values (below) of exact test of population differentiation (100,000 steps).

	Island						
	Palau			Yap			Ngulu
Site	*S*17	*S*20	*S*24	*S*27	*S*29	*S*30	*S*28
*S*17	–	0.05	0.12	0.23	0.16	0.20	0.17
*S*20	0.001	–	0.02	0.12	0.07	0.10	0.09
*S*24	0.001	0.002	–	0.08	0.04	0.07	0.09
*S*27	0.001	0.001	0.001	–	0.09	0.02	0.08
*S*29	0.001	0.001	0.001	0.001	–	0.03	0.10
S30	0.001	0.001	0.001	0.003	0.001	–	0.13
S28	0.001	0.001	0.001	0.001	0.001	0.001	–

#### Bottleneck

To test the hypothesis that *A. hyacinthus* on Palau has recovered from a pulse event of larval dispersal following the 1998 bleaching mortality, we looked for evidence of a recent bottleneck or founder effect using bottleneck 1.2.02 (Piry, Luikart & Cornuet, 1999). In populations that have experienced such an event, rare alleles are the first to be lost, lowering the mean number of alleles per locus. Heterozygosity is less affected, however, producing a transient excess in heterozygosity relative to that expected given the resulting number of alleles (Cornuet & Luikart, 1996). We used the graphical test from Luikart et al. (1998) based on a mode shift away from an L-shaped distribution of allelic frequencies to assess whether evidence of recent population bottlenecks could be detected, which is most appropriate for these types of data (Chaïr et al., 2011). Because we scored fewer than 20 microsatellite loci, we used the Wilcoxon signed-rank test (10,000 iterations) using both a two-phase (TPM incorporated 70% stepwise and 30% multistep mutations) and an infinite allele (IAM) mutational model.

**Figure 3:**
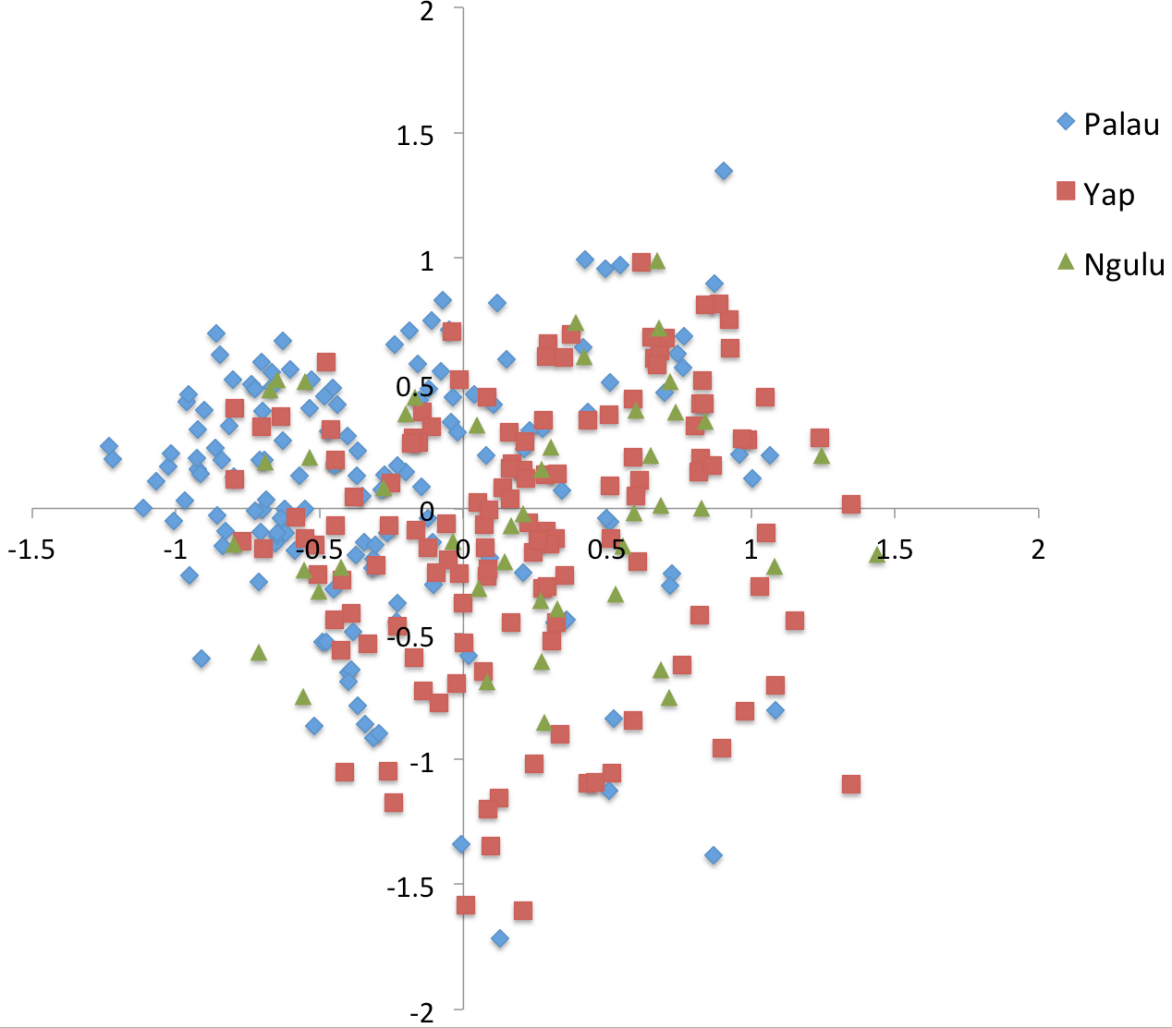
Principal component analysis on individuals for all sites. Results were color coded to show the island at which individuals were found. PCA axis 1 explains 7.2% of the variation and axis 2 explains an additional 5.7%.

**Figure 4:**
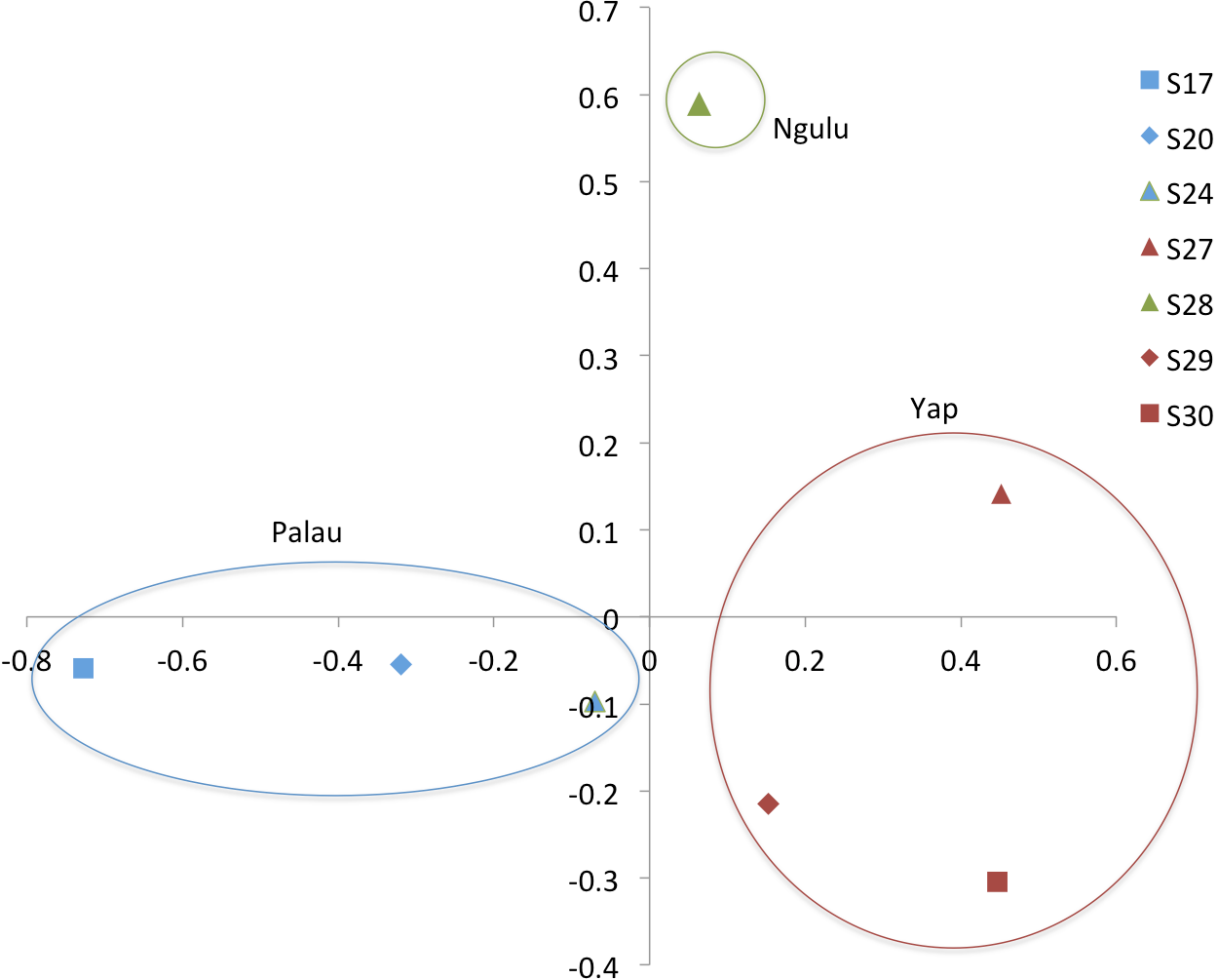
Principal component analysis on sites (populations). The first axis explains 38% of variation and second axis explains 24% of variation.

#### Population structure

A Principal Component Analysis (PCA) was performed in genodive v. 2.0b27 (Meirmans, 2009), both on individuals and on sites using a covariance matrix with 10,000 permutations. The graphs were plotted in Excel (Microsoft, Redmond, Washington, 2010, Figs. 3 and 4).

## Results

### Comparison of datasets

We detected no evidence that alternate strategies of filtering and scoring showed bias in our inference of population differentiation (Table 4). The overall *F*_*ST*_ and *F*′_*ST*_ values as well as the indices of diversity were almost identical for the 10 datasets generated. Genotypes with alleles identified by sequence length gave slightly lower *F*′_*ST*_ and heterozygosity because length masks some of the underlying sequence variation relative to unique IDs, reducing the mean within population heterozygosity and *F*′_*ST*_ (Hedrick, 2005; Jakobsson, Edge & Rosenberg, 2013). Selecting the second allele by depth biases against within population allelic diversity resulted in slightly higher *F*_*ST*_ and *F*′_*ST*_ values and lower *F*_*IS*_ values. As a conservative approach, we selected the first dataset generated by randomly selecting the second allele when there were more than two choices (random000_len). In each case, however, the differentiation among sites was significant (*p* < 0.001), and there are no cases in which inferences would differ because of this variation in magnitude. Because the different strategies for filtering reads produce qualitatively similar results in all cases we believe that the approach of selecting the second allele does not bias our interpretations.

**Table 4:** Comparison of the different datasets created by alternate strategies of filtering and scoring using AMOVA and indices of genetic diversity including number of individuals (*N*), effective number of individuals (*N*_*E*_) expected (*H*_*E*_) and observed (*H*_*O*_) heterozygosities, corrected total heterozygosity (*H*′_*T*_) global *F*_*ST*_ and *F*′_*ST*_ values, significance levels (*p*), and Nei’s corrected fixation index *G*′_*ST*_ and significance level (*p*). Random_000 was generated by randomly selecting the second allele when there were more than two choices; topdepth was generated by selecting the two alleles with the highest number of representation among all colonies, random_min 10 was generated by randomly selecting the second allele of the dataset retaining only alleles that were present in more than 10 colonies, depth_min 10 was generated selecting the two alleles with the highest number of representation among all colonies on the dataset retaining only alleles that were present in more than 10 colonies. Two sets of files were generated: alleles were given a unique identification (_ID) or were identified by their length (_len).

	*N*	*N*_*E*_	*H*_*E*_	*H*_*O*_	*H*′_*T*_	*F*_*ST*_	*F*′_*ST*_	*p*	*F*_*IS*_	*p*	*G*′_*ST*_	*p*
*N* = 321, *Loci* = 11												
random000_ID	27.55	4.95	0.70	0.46	0.73	0.04	0.13	0.001	0.31	0.00	0.04	0.001
topdepth_ID	23.73	4.28	0.67	0.47	0.71	0.05	0.16	0.001	0.25	0.00	0.06	0.001
*N* = 318, *Loci* = 10												
random_min10_ID	12.80	4.20	0.66	0.44	0.70	0.04	0.13	0.001	0.30	0.00	0.05	0.001
depth_min10_ID	12.20	3.62	0.64	0.45	0.68	0.06	0.16	0.001	0.26	0.00	0.06	0.001
*N* = 321, *Loci* = 11												
random000_len	10.40	3.69	0.64	0.43	0.67	0.04	0.11	0.001	0.32	0.00	0.05	0.001
topdepth_len	9.90	3.28	0.62	0.44	0.65	0.06	0.14	0.001	0.27	0.00	0.06	0.001
*N* = 318, *Loci* = 10												
random_min10_len	9.83	3.93	0.67	0.43	0.65	0.04	0.11	0.001	0.31	0.00	0.05	0.001
depth_min10_len	8.00	3.02	0.59	0.42	0.63	0.06	0.14	0.001	0.27	0.00	0.06	0.001

### Population differentiation

Population differentiation was significant among all sites, with a global *F*′_*ST*_ = 0.11. The pairwise differentiation test between islands show that *F*′_*ST*_ values between Yap and Ngulu (0.09), Palau and Ngulu (0.10) and Yap and Palau (0.09) were similar and all comparisons were significant (*p* < 0.01). The exact test between sites (Table 3) shows that both within and among islands, all comparisons are significantly different between Yap, Palau and Ngulu. For example, on Palau, site S24 shows the most differentiation with site S17 (0.12), which is of the same magnitude as comparisons among islands above. We calculated a second measure of genetic differentiation, Jost’s D which gave the same differences between sites (Table S4).

### Bottleneck

The graphical test for bottlenecks, which does not require data to be in Hardy–Weinberg equilibrium, is robust to a small number of loci (fewer than 20) and detects bottlenecks that occurred within a few dozen generations (Luikart et al., 1998). All of the sites had normal L-shaped distributions and the test showed no evidence of bottlenecks for the sites around Palau.

### Population structure

#### Principal component analysis

All samples are distributed more or less evenly along the first and second axis with a slight partition along the first axis such that Palau and Yap sites each cluster together (Fig. 3). Very little of the overall variation is explained, with the first axis explaining only 7.2% and the second axis explaining an additional 5.7% of the variation.

When individuals are grouped by sampling location, sites partition along the first and second axis of the PCA into three quadrants, with Ngulu site S28 standing alone, Palau sites S17, S20 and S24 in the left quadrant and Yap sites S27, S29 and S30 in the right quadrant (Fig. 4). This PCA accounts for 43% of variation along the first axis and the second axis explains an additional 21% of variation.

## Discussion

Connectivity features as a key component of the design of networks of MPAs to increase resilience of both habitats and resources. Larval dispersal and population structure, however, are difficult to measure in the marine environment and different approaches may convey different results to managers. Here, we tested the predictions made by the oceanographic model developed by Golbuu et al. (2012) using high throughput microsatellite genotyping of the coral *A. hyacinthus* and found no evidence that Yap was a significant source of larvae for the recolonization of* A. hyacinthus* after the 1998 bleaching event.

### Microsatellite sequencing

Genotyping microsatellite loci using high throughput sequencing has become cheaper and faster than traditional fluorescent fragment length analysis. To our knowledge, however, there have been very few published papers using this technique (Roberts, Schwartz & Karl, 2004) and no standardized protocol has yet been developed to obtain the best results. The main challenge with sequenced microsatellites is to define a genotype for each colony. There were cases where regardless of the objective filtering criteria applied, more than two alleles were possible, yet corals are diploid organisms. To ensure that our results were robust to decisions about how to select among alternate possible alleles, we tested different methods of allele selection, including selecting two alleles at random from among all sequence variants within an individual. We find that the data are robust to filtering and allele selection criteria, because although the exact values differed, with consistently lower values for selection by depth, none of the indices of heterozygosity, *H*_*O*_, *H*_*E*_, *G*′_*ST*_, or genetic structure, including *F*_*ST*_, *F*′_*ST*_ and *F*_*IS*_, fundamentally changed by using different approaches. This can be explained by previous findings that the greatest portion of the structure and diversity is driven by the most common alleles in the population (Selkoe & Toonen, 2006; Toonen & Grosberg, 2011). This is consistent with our data because neither of the selection criteria impacted the most common alleles in the populations. This robust finding indicates that scoring microsatellites using high throughput technology can give consistent and reliable results that, being based on the underlying sequence rather than length polymorphisms, can allow comparisons of the repeat motif itself and variable flanking regions and be combined reliably among labs and studies. The selection of alleles among several possibilities, however, may prove problematic for applications such as parentage analysis where the genetic pool for parentage assignment will be affected and could lead to false parentage exclusion.

### Connectivity between Palau, Yap and Ngulu

Golbuu et al. (2012) show oceanographic connectivity between Palau and Yap, and argue that the reefs of Palau recovered surprisingly quickly from the mass mortality following the 1998 bleaching event due to a pulse of coral larvae from Yap (including the atoll of Ngulu) in 1999. Bruno et al. (2001) describe populations of *A. hyacinthus* in Palau as having suffered virtually 100% mortality in areas that were surveyed. Given the generation time of *A. hyacinthus* (Wallace, 1985), we would expect roughly three generations in the 14 years between this mortality and our sampling in 2012. If Palau had recovered from a pulse recruitment from Yap after such a widespread and dramatic loss, we would expect evidence of a bottleneck in the recovering population, and should be able to observe a significant founder effect such that Palau would contain a strict subset of the total diversity of Yap and Ngulu from which it was recolonized. Instead, we did not observe signs of a bottleneck, contradicting the hypothesis that *A. hyacinthus* on Palau recovered from a pulse recruitment of larvae from a single source. These data, however, do not exclude the possibility that Palau received a larval pulse from Yap and/or Ngulu, or from more distant populations such as Phoenix Island as demonstrated by Davies et al. (2015), which then mixed with larvae from surviving local populations.

The pairwise comparisons between islands and among sites show similar *F*′_*ST*_ values, highlighting the fact that there is as much differentiation among sites within a single island as among sites on different islands. Although all pairwise *F*′_*ST*_ values are significant (*p* < 0.01) and there is clear population structure among all sites sampled in this study, it is important to note that *F*′_*ST*_ values are small enough to reflect some degree of gene flow through time. This result is more consistent with the prediction of high local retention of larvae in the simulations of Golbuu et al. (2012) than the hypothesized mass recruitment from Yap. Overall, these data support the idea that recovery of *A. hyacinthus* in Palau did not come exclusively from a single source. Instead, the lack of any evidence of a genetic bottleneck and the unique genetic diversity seen in Palau indicate that either mortality was less than 100% and that recovery came from a combination of areas, including sites within Palau. Further, these data indicate that the catastrophic mortality of *A. hyacinthus* reported by Bruno et al. (2001) was likely not as widespread as thought and that there were enough surviving colonies to reseed the barrier reef of Palau as well as maintain genetic diversity.

### Structure of sites within Palau

Sites within Palau separated by as little as 5 km show as much population genetic differentiation as sites between islands separated by as much as 452 km. Both the pairwise and PCA analyses show that Palau’s site S24 (West Palau) is as close to Yap sites S27, S29 and S30 or Ngulu site S28 as it is to Palau site S17 (East Palau). It is interesting to also note that site S17 shows the most differences with other Palau sites S20 and S24 and even greater differences with Yap and Ngulu sites. The eastern lagoon and reef of Palau has historically been impacted by development such as the construction of the airports and roads (Maragos & Cook, 1995) and has been described as being impacted from sediment run-off (Golbuu, 2011; Golbuu et al., 2012). These anthropogenic impacts may have triggered similar population changes to those observed in terrestrial habitats when there is habitat degradation and fragmentation in terrestrial habitats which lead to an erosion of genetic variation and increased genetic divergence between populations due to increased random genetic drift, elevated inbreeding, and reduced gene flow (Young, Boyle & Brown, 1996).

### Implications for conservation

We show that populations of *A. hyacinthus* on Palau did not recover from a single pulse recruitment of larvae, and that mortality was likely less widespread than originally thought, with at least some pockets of surviving colonies within the Palauan archipelago that preserved unique genetic diversity there. Furthermore, there are significant differences among sites around Palau indicating that if there is exchange, it is insufficient to homogenize the populations, supporting the larval dispersal simulations of Golbuu et al. (2012) that there is a high level of self-recruitment among sites. In terms of conservation, these data support increasing the area of conservation by either increasing the number of MPAs or increasing the size of existing MPAs around Palau to protect a wide array of the genetic diversity.

##  Supplemental Information

10.7717/peerj.2330/supp-1 Supplemental Information 1 Tables of genotypic results

10.7717/peerj.2330/supp-2 Figure S1 Genotyping workflow

## Additional Information and Declarations

### Field Study Permissions

The following information was supplied relating to field study approvals (i.e., approving body and any reference numbers): Importation was permitted by Convention on International Trade in Endangered Species of Wild Fauna and Flora #FW 12-091.

### DNA Deposition

The following information was supplied regarding the deposition of DNA sequences: Sequences are provided in Table S1.

### Data Availability

The following information was supplied regarding data availability: DNA sequence data is deposited at the National Center for Biotechnology Information Sequence Read Archive accession number SRP076262. Individual genotype information is available at Dryad http://dx.doi.org/10.5061/dryad.m4q9f.
