# Supplementary material for: Population genetic structure between Yap and Palau for the coral Acropora hyacinthus
Source: PeerJ. 2016 Aug 18;4:e2330. doi: 10.7717/peerj.2330 (PMC4994082; doi:10.7717/peerj.2330)
Supplement: Supplemental Information 1 [file peerj-04-2330-s001.docx]

Supporting Information

Table S1. List of 18 primers amplified, modified from Wang et al. 2008.

| **Primer #** | **Locus** | **Repeat motif** | **Primer sequence (5’-3’)** | **Size (bp)** | **Allel #** | **N** | **No** | **He** | **P** | **Accession #** |
| --- | --- | --- | --- | --- | --- | --- | --- | --- | --- | --- |
| 1 | EST007 | (TTTC)5 | F: TGCAATGGTTCTGTTGCAGTCA  R: GATCTCTTTACCGATTTACAGCA | 99-107 | 3 | 24 | 0.38 | 0.47 | 0.2025 | DY587595 |
| 2 | EST014 | (TCT)13 | F: AGCCGAAGAGGGGACAGAGT  R: AGCCGAAGAGGGGACAGACT | 143-173 | 10 | 24 | 0.92 | 0.88 | 0.6926 | DY586774 |
| 3 | EST016 | (AAC)7 | F: CTATCTGTGTATGATCAGGACTA  R: TCCATCTGTTGTGGAAACTGGT | 97-122 | 7 | 24 | 0.67 | 0.69 | 0.1383 | DY586537 |
| 4 | EST032 | (TTA)21 | F: AGGCACAAGAAAGTGGAAAACAA  R: TGAAGGGATGTGAAGCATGGT | 138-187 | 15 | 24 | 0.96 | 0.94 | 0.7274 | DY585386 |
| 5 | EST062 | (GAT)9 | F: CGAGTTAGTCTGTTAAGATGGT  R: CTCTAAGTCCGATCTTCTTCCA | 110-126 | 5 | 24 | 0.67 | 0.71 | 0.1437 | DY58448 |
| 6 | EST097 | (TGA)7 | F: TGACAACGACATCAATCATGGT  R: ACAGCAGGAGCTGTCAGCACT | 123-135 | 5 | 24 | 0.71 | 0.69 | 0.0923 | DY583334 |
| 7 | EST098 | (TG)12 | F: ACAAATTGCGCTCAAGTTGATG  R: ACGGCTGCGAAGGAGTCTAGT | 98-118 | 8 | 24 | 0.58 | 0.68 | 0.0362 | DY583314 |
| 8 | EST181 | (ATG)10 | F: TGATTGCTGAGAAAGCTAGAGAT  R: GCCTCACCTTGCCTTGTACA | 145-157 | 2 | 24 | 0.25 | 0.22 | 1 | DY580714 |
| 9 | EST196 | (TAA)9 | F: GTGTTGGCTATCTCATGTATAGT  R: ACAACACATCATCAACAACAGCA | 117-145 | 9 | 24 | 0.79 | 0.85 | 0.0926 | DY580091 |
| 10 | EST254 | (CA)10 | F:GGTGACCAATCAGAGTCTTGA  R: TACACTTGCTATAGTAACTTGCT | 86-100 | 8 | 24 | 0.75 | 0.82 | 0.2802 | DY577596 |
| 11 | WGS051 | (GATA)8 | F: GCCGAAACTTCACTGGACGA  R: AAACTTAACTGAGACAACACAGA | 151-216 | 12 | 24 | 0.61 | 0.86 | 0.0004 | 714184394 |
| 12 | WGS092 | (ATT)12 | F: CTGGGCAAATATTACCACTTGA  R: AAGACAGGTATGTATGCAATGAT | 166-184 | 18 | 24 | 0.79 | 0.93 | 0.1645 | 745002572 |
| 13 | WGS112 | (AAT)9 | F: ACTCCACTCAGTCCTATTACCA  R: ACACTTCCAAGAGTCCCTACA | 166-184 | 6 | 24 | 0.79 | 0.73 | 0.9619 | 745001340 |
| 14 | WGS134 | (GATA)6 | F: TGTTCGGACCCCAACCTGAT  R: GCTGCGCCCTTCGCAATTCA | 105-133 | 7 | 24 | 0.58 | 0.67 | 0.3767 | 745001492 |
| 15 | WGS152 | (AT)9 | F: GCCTATTTACAATGCATAGCACTA  R: CGCTGGGTCCTATCTATATCT | 98-118 | 7 | 24 | 0.54 | 0.76 | 0.0241 | 714180564 |
| 16 | WGS153 | (AATC)7 | F: TTTCCAAGTTGCTGTGAGTACA  R: CGCTGGGTCCTATCTATATCT | 106-126 | 5 | 24 | 0.63 | 0.64 | 0.9458 | 714176682 |
| 17 | WGS189 | (ATCT)7 | F: AAATGAGCGCCTGTGCACGA  R: GAGCATGAAACTCTGAGTAGCA | 158-194 | 9 | 24 | 0.58 | 0.75 | 0.0420 | 714180544 |
| 18 | WGS211 | (TAA)8 | F: TGACGACGAAACGTTGGCTAT  R: AGACCGTTTCCTTTAACCAGAA | 181-199 | 5 | 24 | 0.75 | 0.61 | 0.4021 | 714178565 |

Tabl;e S2. Example of tags added to the forward primers as a colony ID #1 t #48 for primer 1, locus EST007, and microsatellite TTTC.

| ID number | Tag + Primer Sequence | Tag |
| --- | --- | --- |
| 1 | AACACCTGCAATGGTTCTGTTGCAGTCA | AACACC |
| 2 | GTTAGGTGCAATGGTTCTGTTGCAGTCA | GTTAGG |
| 3 | AACGGATGCAATGGTTCTGTTGCAGTCA | AACGGA |
| 4 | AAGAGGTGCAATGGTTCTGTTGCAGTCA | AAGAGG |
| 5 | GTTCCATGCAATGGTTCTGTTGCAGTCA | GTTCCA |
| 6 | TATGCGTGCAATGGTTCTGTTGCAGTCA | TATGCG |
| 7 | TCCTTGTGCAATGGTTCTGTTGCAGTCA | TCCTTG |
| 8 | ACAACGTGCAATGGTTCTGTTGCAGTCA | ACAACG |
| 9 | TCGACTTGCAATGGTTCTGTTGCAGTCA | TCGACT |
| 10 | ACCTCATGCAATGGTTCTGTTGCAGTCA | ACCTCA |
| 11 | ACGCAATGCAATGGTTCTGTTGCAGTCA | ACGCAA |
| 12 | ACGTGTTGCAATGGTTCTGTTGCAGTCA | ACGTGT |
| 13 | ACTCTGTGCAATGGTTCTGTTGCAGTCA | ACTCTG |
| 14 | TGACCATGCAATGGTTCTGTTGCAGTCA | TGACCA |
| 15 | AGCATGTGCAATGGTTCTGTTGCAGTCA | AGCATG |
| 16 | TGGAAGTGCAATGGTTCTGTTGCAGTCA | TGGAAG |
| 17 | AGGACATGCAATGGTTCTGTTGCAGTCA | AGGACA |
| 18 | TGGTGATGCAATGGTTCTGTTGCAGTCA | TGGTGA |
| 19 | ATGTCCTGCAATGGTTCTGTTGCAGTCA | ATGTCC |
| 20 | CAACTCTGCAATGGTTCTGTTGCAGTCA | CAACTC |
| 21 | CAAGCATGCAATGGTTCTGTTGCAGTCA | CAAGCA |
| 22 | CAATGGTGCAATGGTTCTGTTGCAGTCA | CAATGG |
| 23 | CACAGTTGCAATGGTTCTGTTGCAGTCA | CACAGT |
| 24 | TTGGCATGCAATGGTTCTGTTGCAGTCA | TTGGCA |
| 25 | CCATACTGCAATGGTTCTGTTGCAGTCA | CCATAC |
| 26 | CCGTTATGCAATGGTTCTGTTGCAGTCA | CCGTTA |
| 27 | AGACTTGCAATGGTTCTGTTGCAGTCA | AGACT |
| 28 | CCTTCTTGCAATGGTTCTGTTGCAGTCA | CCTTCT |
| 29 | CGAGTTTGCAATGGTTCTGTTGCAGTCA | CGAGTT |
| 30 | CGTAGATGCAATGGTTCTGTTGCAGTCA | CGTAGA |
| 31 | ATCTGTGCAATGGTTCTGTTGCAGTCA | ATCTG |
| 32 | CTACCTTGCAATGGTTCTGTTGCAGTCA | CTACCT |
| 33 | CTCACATGCAATGGTTCTGTTGCAGTCA | CTCACA |
| 34 | CTGAACTGCAATGGTTCTGTTGCAGTCA | CTGAAC |
| 35 | CTTGGTTGCAATGGTTCTGTTGCAGTCA | CTTGGT |
| 36 | AAGCGTGCAATGGTTCTGTTGCAGTCA | AAGCG |
| 37 | GACTTCTGCAATGGTTCTGTTGCAGTCA | GACTTC |
| 38 | GAGTCATGCAATGGTTCTGTTGCAGTCA | GAGTCA |
| 39 | ATTCCTGCAATGGTTCTGTTGCAGTCA | ATTCC |
| 40 | GCACTATGCAATGGTTCTGTTGCAGTCA | GCACTA |
| 41 | GCCATTTGCAATGGTTCTGTTGCAGTCA | GCCATT |
| 42 | GCTTGATGCAATGGTTCTGTTGCAGTCA | GCTTGA |
| 43 | CACCTTGCAATGGTTCTGTTGCAGTCA | CACCT |
| 44 | GGATCTTGCAATGGTTCTGTTGCAGTCA | GGATCT |
| 45 | GTAACCTGCAATGGTTCTGTTGCAGTCA | GTAACC |
| 46 | GTACAGTGCAATGGTTCTGTTGCAGTCA | GTACAG |
| 47 | GTCGTATGCAATGGTTCTGTTGCAGTCA | GTCGTA |
| 48 | GTGCTTTGCAATGGTTCTGTTGCAGTCA | GTGCTT |

| Locus | # Alleles | Heterozygosity (SD)  random000_len | Heterozygosity (SD)  random000_ID | Length Range (nt) |
| --- | --- | --- | --- | --- |
| Locus 1 | 2 | 0.34±0.10 | 0.35±0.10 | 28-36 |
| Locus 3 | 6 | 0.69±0.07 | 0.71±0.08 | 21-36 |
| Locus 4 | 20 | 0.91±0.02 | 0.92±0.02 | 15-75 |
| Locus 5 | 11 | 0.55±0.05 | 0.63±0.06 | 57-90 |
| Locus 6 | 4 | 0.50±0.11 | 0.57±0.14 | 36-45 |
| Locus 8 | 14 | 0.83±0.03 | 0.90±0.03 | 39-108 |
| Locus 11 | 8 | 0.66±0.06 | 0.79±0.02 | 12-44 |
| Locus 12 | 17 | 0.83±0.05 | 0.91±0.06 | 36-93 |
| Locus 13 | 8 | 0.72±0.08 | 0.79±0.10 | 54-81 |
| Locus 14 | 12 | 0.68±0.07 | 0.74±0.07 | 20-84 |
| Locus 16 | 7 | 0.32±0.24 | 0.3±0.25 | 32-56 |

Table S3. Number of alleles, heterozygosity values with standard deviation for datasets random000_len and random000_ID and range of length of microsatellites for each locus.

Table S4. Jost’s D pairwise differentiation.

|  |  | Island | | | | | | | | |
| --- | --- | --- | --- | --- | --- | --- | --- | --- | --- | --- |
|  |  | Palau | | |  | Yap | | |  | Ngulu |
| Site |  | S17 | S20 | S24 |  | S27 | S29 | S30 |  | S28 |
| S17 |  | -- | 0.04 | 0.09 |  | 0.16 | 0.11 | 0.16 |  | 0.12 |
| S20 |  |  | -- | 0.02 |  | 0.09 | 0.05 | 0.08 |  | 0.07 |
| S24 |  |  |  | -- |  | 0.07 | 0.04 | 0.08 |  | 0.08 |
| S27 |  |  |  |  |  | -- | 0.07 | 0.03 |  | 0.06 |
| S29 |  |  |  |  |  |  | -- | 0.03 |  | 0.07 |
| S30 |  |  |  |  |  |  |  | -- |  | 0.11 |
| S28 |  |  |  |  |  |  |  |  |  | -- |
