# Supplementary figures and images for: Population genetic structure between Yap and Palau for the coral Acropora hyacinthus
Source: PeerJ. 2016 Aug 18;4:e2330. doi: 10.7717/peerj.2330 (PMC4994082; doi:10.7717/peerj.2330)

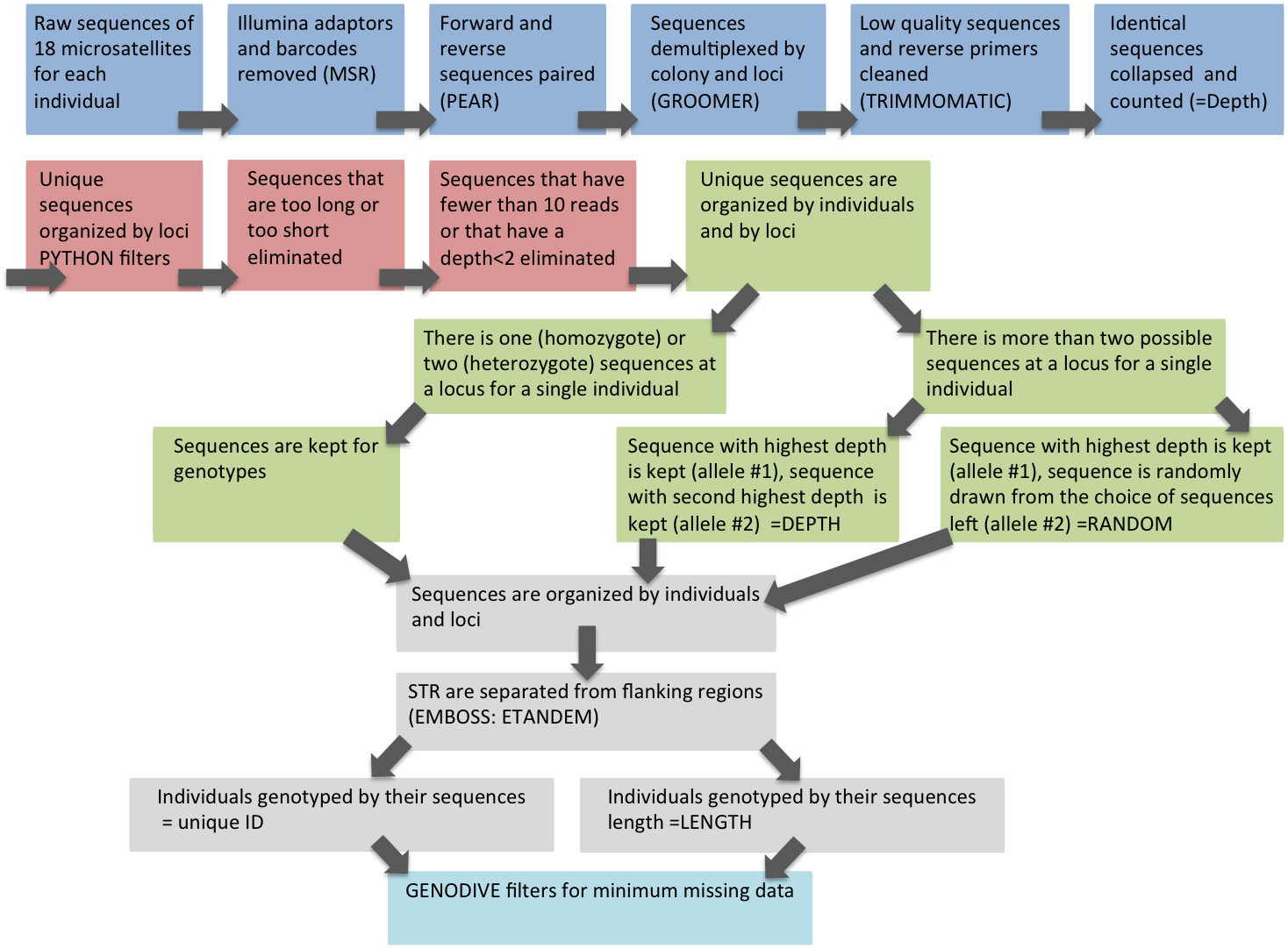

Supplement: Figure S1 [file peerj-04-2330-s002.png]
